# Dr. Frank L. Sutton

**Published:** 1912-03

**Authors:** 


Dr. Frank L. Sutton College of Physicians and surgeons (Homeopathic, Buffalo) 1822, for many years a practitioner in Canisteo, N. Y., died at his home in Port Allegany, Pa., from heart disease, Jan. 7, 1912, aged 57.

